# Effects of kilohertz versus low‐frequency electrical stimulation of the wrist extensors in patients after stroke: A randomized crossover trial

**DOI:** 10.1002/pmrj.13368

**Published:** 2025-05-13

**Authors:** Sarah Tenberg, Jonas Weinig, Daniel Niederer, Lutz Vogt, Markus Leisse, Steffen Müller

**Affiliations:** ^1^ Department of Computer Science/Therapeutic Sciences Trier University of Applied Sciences Trier Germany; ^2^ Department of Sports Medicine and Exercise Physiology Goethe‐University Frankfurt Frankfurt am Main Germany; ^3^ Institute of Occupational, Social and Environmental Medicine, Department of Sports Medicine and Exercise Physiology Goethe University Frankfurt Frankfurt am Main Germany; ^4^ MEDIAN Rehabilitation Centre, Klinik Burg Landshut Bernkastel‐Kues Germany

## Abstract

**Background:**

Electrical stimulation is an effective treatment method for improving motor function after stroke, but the optimal current type for patients with stroke and arm paresis remains unclear.

**Objective:**

To compare the effects of kilohertz frequency with low‐frequency current on stimulation efficiency, electrically induced force, discomfort, and muscle fatigue in patients with stroke.

**Design:**

A randomized crossover study.

**Setting:**

Neurological inpatient rehabilitation clinic in Germany.

**Participants:**

A total of 23 patients with arm paresis after stroke within the last 6 months were recruited, 21 were enrolled, and 20 completed the study (7 females; mean ± SD: 66 ± 12 years; 176 ± 11 cm; 90 ± 19 kg; 57 ± 34 days since stroke).

**Intervention:**

All patients underwent both kilohertz and low‐frequency stimulation in a randomized order on 2 days (48‐hour washout). Each day included a step protocol with a gradual increase in stimulation intensity, starting at the first measurable force (up to 12 steps, 1 mA increments, 8 seconds stimulation, 60 second rest) and a fatigue protocol (30 repetitions, 8 second stimulation, 3 second rest).

**Main Outcome Measure:**

Primary outcome was stimulation efficiency (electrically induced force/stimulation intensity) [N/mA], measured during each step of the stepwise increase in current intensity protocol.

**Results:**

Linear‐mixed‐effects models showed significantly higher stimulation efficiency for low‐frequency stimulation (mean difference 0.14 [95% confidence interval, 0.01–0.27 N/mA], *p* = .031). However, current type did not significantly affect electrically induced force, level of discomfort, or muscle fatigue (*p* > .05).

**Conclusion:**

The findings suggest that low‐frequency stimulation is more efficient than kilohertz‐frequency stimulation. However, both current types yield similar effects on force, discomfort, and fatigue, making them both viable options for wrist extensor stimulation in patients after stroke. Considering the variability among individuals, customizing the current type based on electrically induced force and perceived discomfort may enhance therapeutic outcomes. Further research on the long‐term treatment effects of both current types is warranted.

## INTRODUCTION

Stroke, the third leading cause of combined mortality and disability‐adjusted life years lost worldwide,^1^, ^2^ results in upper limb impairments in approximately 77% of survivors.^3^ Arm paresis in particular has a significant impact on daily life activities.^4^ Notably, cyclic and especially functional electrical stimulation have shown great benefit on strength and activity performance in patients with stroke.^5^, ^6^, ^7^, ^8^, ^9^, ^10^

For an effective application of electrical stimulation in clinical settings, maximizing force output is crucial.^11^, ^12^ However, this depends on the stimulation intensity, which can be increased according to the patient's tolerance.^12^ In addition, the rapid onset of muscle fatigue caused by electrical stimulation represents a major limitation.^11^ For appropriate electrical stimulation in patients with stroke with arm paresis, the stimulation should be effective (resulting in high force output relative to the stimulation intensity) and causing minimal discomfort and muscle fatigue.

Given the various configuration possibilities of electrical stimulation, researchers are investigating the effects of different electrical stimulation parameters, specifically comparing low‐frequency and kilohertz frequency stimulation in healthy participants. Findings suggest that 2.5 kilohertz frequency stimulation with narrow pulse duration yields comparable or lower level of force, comparable discomfort, comparable current efficiency for the maximum tolerated stimulation intensity, and greater fatigue when compared to low‐frequency stimulation.^13^ Conversely, 1.0 kilohertz frequency stimulation with wide pulse duration (500 μs) elicited no differences in torque production when compared to a low‐frequency stimulation with wide pulse duration.^14^, ^15^, ^16^ In addition, the 1.0 kilohertz frequency stimulation was associated with less discomfort in the wrist extensors^14^ and stimulation efficiency (electrically induced force/stimulation intensity) was improved with wide phase durations regardless of carrier frequency.^15^ Studies examining the efficiency of wrist stimulation and the fatiguing effect of the 1.0 kilohertz frequency are lacking. It is also uncertain whether these findings are transferable to patients after stroke. Given the complexity of the neurological picture of stroke and the heterogeneity of patient characteristics, current types should be compared at the individual level.

Therefore, this randomized crossover study aimed to investigate the acute effects of a 1.0 kilohertz frequency versus a low‐frequency electrical stimulation of the wrist extensors on (1) stimulation efficiency (force/stimulation intensity), (2) force production, (3) perceived discomfort, and (4) muscle fatigue in individuals with stroke and arm paresis at submaximal stimulation intensity. We hypothesized that a 1.0 kilohertz frequency stimulation results in comparable stimulation efficiency, force production, and muscle fatigue, as well as less discomfort, compared to low‐frequency stimulation.

## MATERIALS AND METHODS

### Study design and ethics

The trial adopted a block‐randomized single‐blind crossover design. The study was approved by the local ethics committee of the University of Applied Sciences Trier (No. 01‐2023), conducted in compliance with the Declaration of Helsinki and was registered a priori in the German Clinical Trials Register (DRKS‐ID: DRKS00030666). The reporting of the study was made according to the Consolidated Standards of Reporting Trials extension statement for crossover trials.^17^ The completed checklist can be found in Table A1.

### Role of the funding source

This research received funding from the German Federal Ministry for Economic Affairs and Energy. The funder played no role in the design, conduct, or reporting of this study.

### Participants

Participants were recruited from a neurological rehabilitation clinic in Germany between March and November 2023. Participants were informed about the study, agreed to participate, and provided verbal and written informed consent.

Inclusion criteria were hemorrhagic or ischemic stroke with arm paresis in the previous 6 months and being at least 18 years of age. Arm paresis was indicated by a wrist extensor muscle function between 1 and 4 according to Janda.^18^ In addition, participants had to be medically cleared for the study by the treating neurologist.

Participants with contraindications to electrical stimulation according to Wenk^19^ were excluded from the study. Additionally, patients who were unable to sit for at least 60 minutes or who had cognitive impairments hindering their understanding of the study procedures were excluded. Participants who were unable to tolerate the intensity of electrical stimulation for wrist extension were also excluded.

Sample size was determined a priori using G*Power (version 3.1; University of Kiel, Germany) with a significance level of *p* < .05 and a power at (1−*β*) of 0.8. Sample size was determined using the reliability data (5.96 N) (ie, SEM) and the sample SD (13.5 N) of the dependent variable electrically induced force from healthy participants.^20^ Based on the calculated effect size of Cohen's d = 0.57, 21 participants were defined as the minimum number for the study. With a potential dropout rate of 10%–20%, the aim was to recruit 24 participants for the study.

### Randomization and blinding

The two measurement conditions, 1.0 kilohertz frequency and low‐frequency electrical stimulation, were randomized using a computer generated randomization list in R studio (Version 4.1.0, Boston, MA, USA). A permuted block randomization with a block length of eight was used. The randomization list was generated by S.T. The recruiting physician was not aware of the randomization method and sequence. S.T. and J.W. were aware of the randomization method and the list and assigned the patients accordingly on the familiarization day. Participants were blinded to current type and stimulation intensity. The investigators (S.T. and J.W.) who applied the electrical stimulation were not blinded to be able to control the stimulation intensity.

### Electrical stimulation

**FIGURE 1:**
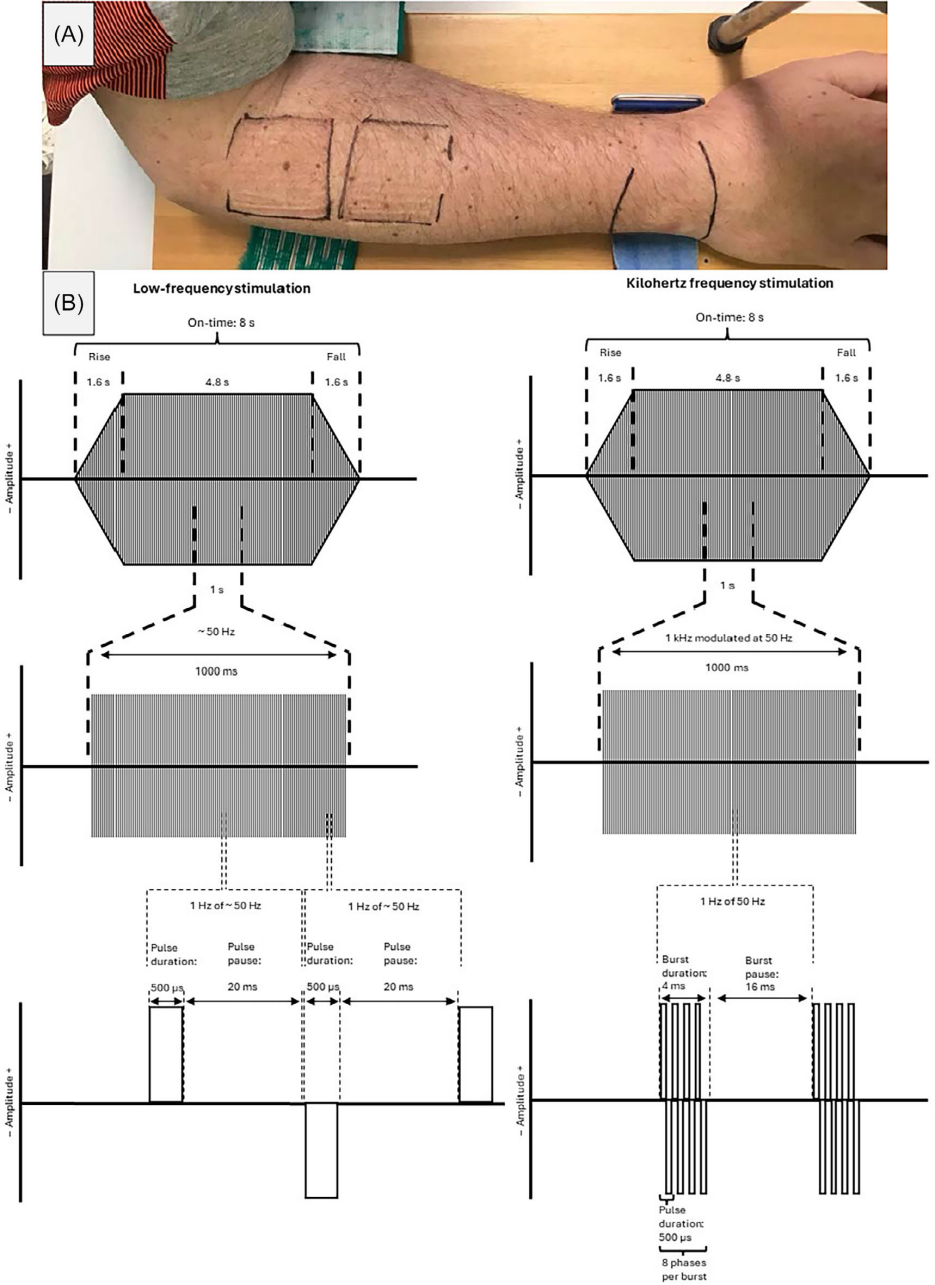
(A) Marking of electrode positions and forearm attachment for the force measurement on a participant's skin. (B) Schematic description of pulse delivery for low‐frequency (left) and kilohertz frequency stimulation (right). Illustration adapted from previous work of Paz et al.^26^

The PHYSIODYN‐Expert device PHYSIOMED ELEKTROMEDIZIN AG, Germany) was used for the application of electrical stimulation. Two plate electrodes with molded sponges containing approximately 12 g of water and measuring 4 × 4 cm were used for stimulation. These were positioned proximally with one electrode distal to the lateral epicondyle of the humerus and the second one 15 mm distal of the first electrode (Figure 1A).^21^ The electrode positions were marked on the skin with a waterproof pen after the first measurement session to be able to reposition them as close as possible for the second measurement session.

For both current types, biphasic, symmetrical rectangular pulses and a ramp of 20%/60%/20% of the total contraction time were used. Low‐frequency stimulation was applied with a pulse duration of 500 μs and a pulse pause of 20 ms, resulting in approximately 50 Hz. Kilohertz frequency stimulation was applied at 1 kilohertz, burst modulated to 50 Hz with a burst duration of 4 ms and a burst pause of 16 ms and a pulse duration of 500 μs. The participants were asked to relax during the electrical stimulation protocols. A schematic description of the current types can be found in Figure 1B.

### Study procedure

The study consisted of two sessions of electrical stimulation of the wrist extensors using a kilohertz and a low‐frequency stimulation. Each session lasted about 1 hour with a 48‐hour washout period between sessions. Participants were tested at the same rehabilitation center, by the same investigator, at the same time of the day. Participants were familiarized with the procedure in an a priori session. Measurement sessions comprised a protocol of stepwise increase in current intensity and a protocol of muscle fatigue.

#### Familiarization session

Participants were asked about their demographics and health condition. Prestroke handedness was assessed with the Edinburgh Handedness Inventory Short Form.^22^ Muscle function assessments were made according to Janda.^18^ Lastly, sensation of touch and pressure was tested by checking whether the participant could perceive a touch in the area of the electrode placement on the muscle belly of the musculus extensor carpi radialis brevis with the eyes closed, and testing the pressure pain threshold with three repetitions and a pause of 30 seconds in between.^23^ The mean of three repetitions was defined as the pressure pain threshold. Finally, the length of the lever arm of the hand was measured from the articular space of the wrist to caput metacarpi III.

Participants were familiarized with electrical stimulation using the randomized order of current types. The sensory threshold (the first sensory response when the current is noticeable)^24^ was determined, followed by 10 repetitions at that intensity. The same procedure was repeated with the intensity for the motor threshold (first motor response, eg, muscle twitch)^24^ and for the intensity required for maximal wrist extension, as determined by the investigator. The same procedure was repeated for the second current type.

**FIGURE 2:**
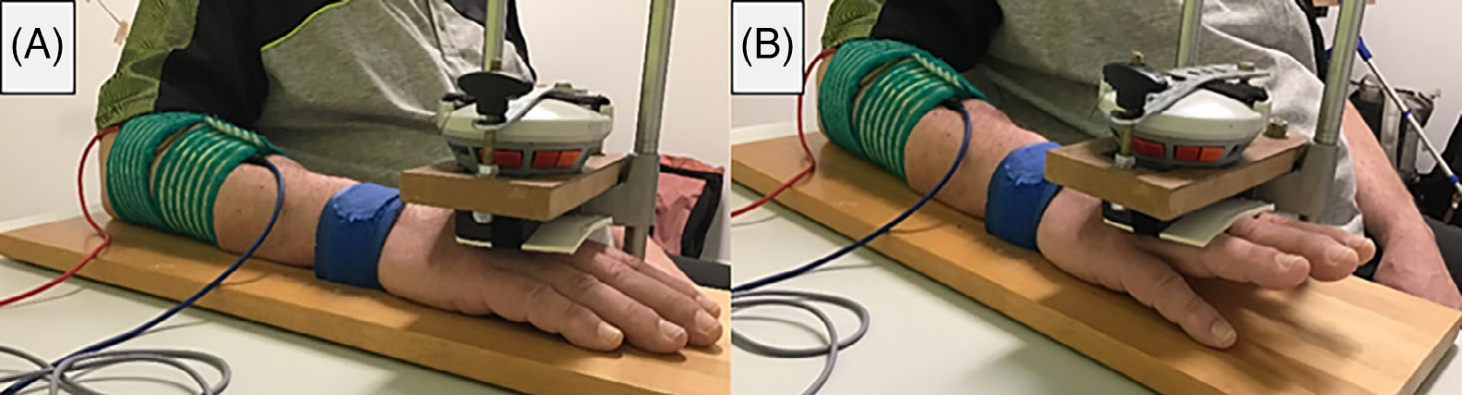
Measurement of the electrically induced force by means of the hand‐held dynamometer attached to the customized measurement construct with (A) the starting position, no stimulation and (B) stimulation with force measurement.

Maximum voluntary isometric contraction (MVIC) of the unaffected hand was measured in Newton [N] using a hand‐held dynamometer (microFET 2, Hoggan Scientific LLC, USA) attached to a customized and validated measurement construct at approximately 10 degrees of wrist extension (Figure 2). The reliability of the device was previously tested on healthy control participants.^20^ In particular, the MVIC measurement showed excellent reliability (intraclass correlation coefficient, 0.978 [95% confidence interval (CI), 0.929–0.994]). A warmup consisting of 10 dynamic wrist extension and flexion movements, an isometric contraction of approximately 50% and 80% of maximal force, was performed, followed by three maximal voluntary isometric wrist extension contractions with 60‐s rest in between. The mean of the three repetitions was defined as MVIC.

#### Measurement sessions

Both measurement sessions were identical except for the current type. The three electrical stimulation threshold tests were repeated to reaccustom the patient and verify electrode placement. MVIC of the affected hand was measured using the same procedure as for the unaffected hand. A stepwise increase in stimulation intensity protocol and a muscle fatigue protocol were then performed, with a 5‐minute rest in between.

For the modified step protocol,^15^ the intensity of the first measurable force determined the first stimulation step. Intensity was then increased in 1 mA increments for a total of 12 stimulation steps, as tolerated. The stimulation duration was 8 s with a 60‐s rest between contractions.

The modified muscle fatigue protocol^25^ involved 30 contractions at a standardized current intensity (first measurable force * 1.25) each lasting 8 s with 3‐s rest intervals.

### Outcomes

The primary outcome was stimulation efficiency, calculated as the quotient of electrically induced force and stimulation intensity (N/mA),^15^, ^16^, ^26^ and measured for each step of the stepwise increase in stimulation intensity protocol.

Secondary outcomes included electrically induced force, perceived discomfort, and muscle fatigue. Electrically induced force was measured in [N] at each step/repetition of the stepwise increase in stimulation intensity protocol and the muscle fatigue protocol using the previously described measurement construct and position (Figure 2).

Discomfort, rated on a numerical rating scale from 0 (= not at all uncomfortable) to 10 (= maximum discomfort imaginable), was assessed at each repetition of the stepwise increase in current intensity protocol. Muscle fatigue was determined for the muscle fatigue protocol and defined as the decrease in electrically induced force [N] during the muscle fatigue protocol.

In addition, the muscle fatigue index (first five electrically induced force values/last five force values), relative force (mean of the last five repetitions in relation to the first five repetitions as a percentage) and total force during the muscle fatigue protocol were calculated.

**FIGURE 3:**
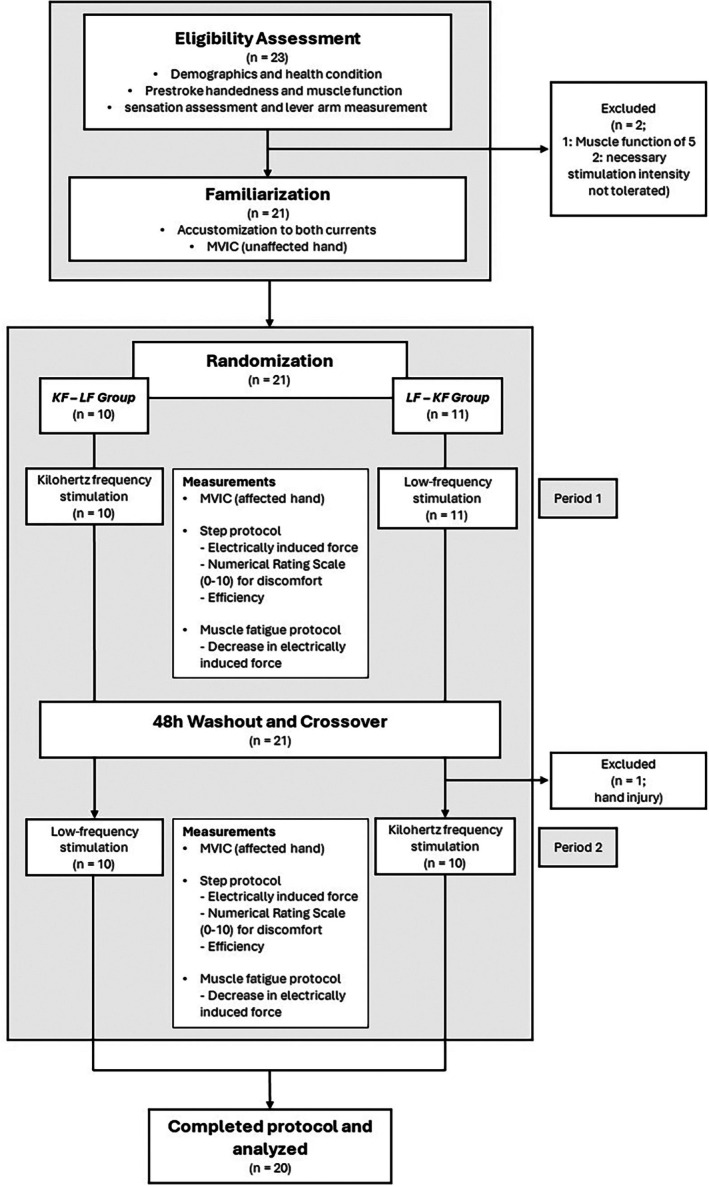
Flow chart of the study procedure and participant flow. efficiency, electrically induced force/stimulation intensity; KF‐LF group, participants who started with kilohertz frequency stimulation followed by low‐frequency stimulation; F‐KF group, participants who started with low‐frequency stimulation followed by kilohertz frequency stimulation; MVIC, maximal voluntary isometric contraction.

The complete study procedure with the corresponding outcome assessments is shown in Figure 3.

### Statistical analyses

To investigate a possible carryover effect, carryover tests were performed according to Wellek and Blettner^27^ for the primary outcome (stimulation efficiency) at each step of the stepwise increase of current intensity protocol and for the mean electrically induced force of the first five and last five repetitions of the muscle fatigue protocol.

Data are presented descriptively as number of participants, median (range), or mean ± SD.

For inferential statistics, linear mixed‐effects models with restricted maximum likelihood estimation were used to analyze stimulation efficiency and discomfort progression during steps of the stepwise increase of stimulation intensity protocol between current types and participants and electrically induced force progression during steps/repetitions for both protocols. Step or repetition, along with current type were considered fixed effects, whereas participants were considered random effects. Main and interaction effects were taken into account in the models. Covariates (absolute and relative MVIC, stroke type, time since stroke, muscle function, weight, height, age, pressure pain threshold, gender, dominant hand affected, and lever arm) were integrated as fixed effects using a stepwise approach. The 11 covariates were sequentially added, and model fit was identified using Akaike's information criterion (AIC). The model with the covariates resulting in the lowest AIC was selected for the covariate analysis. For random effects, variance and SD were calculated. For fixed effects, estimate (*e*), 95% CI, *T*‐statistics (*t*), and significance level (*p*) were calculated. The level of significance was set, a priori, to *α* = .05. Intraclass correlation coefficient and marginal (variance clarification through fixed effects) and conditional (variance clarification through random and fixed effects) *r*‐squared (*r* ^2^) were calculated for the model. LmerTest package in R (version 4.1.0, Boston, MA, USA) was used for linear‐mixed‐effects model analysis.

## RESULTS

In total, 20 participants completed the study and could be included in the analyses. Participant flow diagram can be found in Figure 3. The characteristics of the participants are shown in Table 1. Descriptive results for the different current types can be found in Table 2.

**TABLE 1:** Characteristics of the study population, separated by sequence groups.

Characteristics	LF‐KF group (starting with low frequency)	KF‐LF group (starting with kilohertz frequency)	Total
Number (*n*)	10	10	20
Male/female (*n*)	5/5	8/2	13/7
Age (years)	62 (7)	69 (15)	66 (12)
Height in cm	176 (13)	175 (10)	176 (11)
Weight in kg	93 (15)	87 (23)	90 (19)
Days since stroke	49 (27)	63 (40)	57 (34)
Ischemic/hemorrhagic/unknown (*n*)	10/0/0	6/3/1	16/3/1
Muscle function according to Janda (median [minimum−maximum])	3.5 (1–4)	3 (1–4)	3 (1–4)
Maximal voluntary isometric contraction of the affected hand in Newton	45 (29)	55 (30)	50 (29)
Maximal voluntary isometric contraction of the unaffected hand in Newton	97 (30)	101 (36)	99 (33)
Affected hand dominant/nondominant (*n*)	4/6	6/4	10/10
Pressure pain threshold in kPa	1.8 (0.8)	2.1 (0.4)	1.9 (0.6)
Lever arm of the affected hand in cm	8.1 (0.6)	8.0 (0.6)	8.0 (0.6)

*Note*: Data are displayed as mean (standard deviation) unless stated otherwise. Abbreviations: KF‐LF group, participants who started with kilohertz frequency stimulation followed by low‐frequency stimulation; kPA, kilopascal; LF‐KF group, participants who started with low‐frequency stimulation followed by kilohertz frequency stimulation.

**TABLE 2:** Results of the comparison of the two different current types pooled for both sequence groups.

	Low frequency	Kilohertz frequency
Highest stimulation efficiency [N/mA]	1.22 (0.79)	0.90 (0.53)
Highest electrically induced force [N]	32 (18)	31 (18)
Highest discomfort level (numerical rating scale, 0–10)	4 (3)	5 (4)
Relative force at the end of the muscle fatigue protocol (%)	68 (21)	70 (38)
Fatigue index (electrically induced force mean of the first five repetitions/mean of the last five repetitions)	1.77 (1.24)	2.05 (1.69)
Total force (sum of all repetitions during the muscle fatigue protocol) [N] [median (minimum to maximum])	334 (116 to 1661)	356 (120 to 1116)
Highest stimulation intensity at last step [mA]	29 (5)	40 (9)
Stimulation steps completed (number of steps)	11.5 (1)	12 (1)

*Note*: Data are presented as mean±SD unless stated otherwise. Abbreviations: mA, milliampere; N, Newton; stimulation efficiency, electrically induced force/stimulation intensity.

No carryover effect was detected using the Mann–Whitney *U* test (*p* > .05).

### Stimulation efficiency [N/mA] (primary outcome)

**FIGURE 4:**
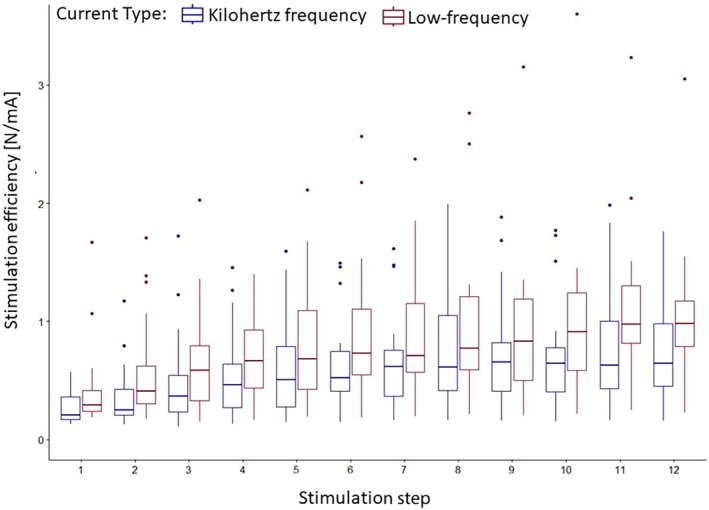
Stimulation efficiency (electrically induced force/stimulation intensity) [N/mA] during the stepwise increase in stimulation intensity protocol for both current types. Data are displayed as boxplots (median, interquartile ranges, and whisker bars).

Linear‐mixed‐effects model revealed intercept (*p* < .001), current type (0.031), and stimulation step (*p* < .001) as significant predictors for the primary outcome stimulation efficiency. In addition, an interaction effect was found for step*current type (*p* = .003). On average, low‐frequency stimulation exhibited 0.14 (0.01–0.27) [N/mA] higher efficiency compared to kilohertz frequency stimulation. Efficiency improved by an average of 0.05 (0.03–0.07) [N/mA] per stimulation step and additionally 0.01 (0.01–0.02) [N/mA] improvement per step for low‐frequency stimulation. The primary sources of variance were attributed to the interaction between participant and current type (0.06 ± 0.25 N/mA), followed by individual participant differences (0.04 ± 0.19 N/mA). Full results can be found in Table 3. The descriptive results can be found in Table 2 and Figure 4.

**TABLE 3:** Linear mixed effects model results.

Participants = 20	Efficiency (electrically induced force/stimulation intensity) [N/mA]	Electrically induced force [N]	Discomfort (numerical rating scale, 0–10)	Muscle fatigue (decrease in electrically induced force) [N]
Fixed effects	Estimates (CI), *t*, *p*	Estimates (CI), *t*, *p*	Estimates (CI), *t*, *p*	Estimates (CI), *t*, *p*
(Intercept)	0.32 (0.22 to 0.41), *t* = 6.39, ***p <* .001**	6.88 (4.44 to 9.33), *t* = 5.54, ***p* < .001**	1.83 (0.96 to 2.69), *t* = 4.15, ***p* < .001**	16.76 (12.21 to 21.31), *t* = 7.23, ***p* < .001**
Step	0.05 (0.03 to 0.07), *t* = 5.15, ***p* < .001**	2.17 (1.52 to 2.81), *t* = 6.58, ***p* < .001**	0.24 (0.15 to 0.34), *t* = 4.94, ***p* < .001**	−0.22 (−0.32 to −0.13), *t* = −4.58, ***p* < .001**
Current type	0.14 (0.01 to 0.27), *t* = 2.17, ***p* = .031**	−1.35 (−4.27 to 1.56), *t* = −0.91, *p* = .362	−0.22 (−0.71 to 0.27), *t* = −0.88, *p* = .379	1.91 (−2.13 to 5.94), *t* = 0.93, *p* = .354
Step: CurrentType	0.01 (0.01 to 0.02), *t* = 3.03, ***p* = .003**	0.17 (−0.07 to 0.41), *t* = 1.38, *p* = .170	0.01 (−0.02 to 0.03), *t* = 0.44, *p* = .661	0.02 (−0.03 to 0.07), *t* = 0.72, *p* = .469
Random effects	Variance ± SD	Variance ± SD	Variance ± SD	Variance ± SD
σ^2^	0.03 ± 0.18	19.84 ± 4.45	0.28 ± 0.53	13.18 ± 3.63
τ_00 Participant_	.04 ± 0.19	23.28 ± 4.83	3.77 ± 1.94	105.55 ± 10.27
τ_11 Participant. Step_	0.001 ± 0.04	2.02 ± 1.42	0.05 ± 0.22	0.04 ± 0.20
τ_11 Participant.CurrentType low‐frequency_	0.06 ± 0.25	28.75 ± 5.36	1.04 ± 1.02	80.89 ± 9.00
Mixed model information	ICC, observations Marginal *R* ^2^/conditional *R* ^2^	ICC, observations Marginal *R* ^2^/conditional *R* ^2^	ICC, observations Marginal *R* ^2^/conditional *R* ^2^	ICC, observations Marginal *R* ^2^/conditional *R* ^2^
	0.87, 468 0.170/0.893	0.89, 468 0.250/0.916	0.97, 468 0.083/0.968	0.90, 1194 0.034/0.902

Abbreviations: CI, confidence interval; ICC, intraclass correlation coefficient; mA, milliampere; N, Newton; *R* ^2^, variance clarification through fixed (marginal), and fixed and random effects (conditional); *t*, *t*‐statistic; σ^2^, residual.

For the covariate analysis, MVIC and stroke type were included in the model, with MVIC showing a significant effect (*p* = .005).

### Electrically induced force [N] (secondary outcome)

**FIGURE 5:**
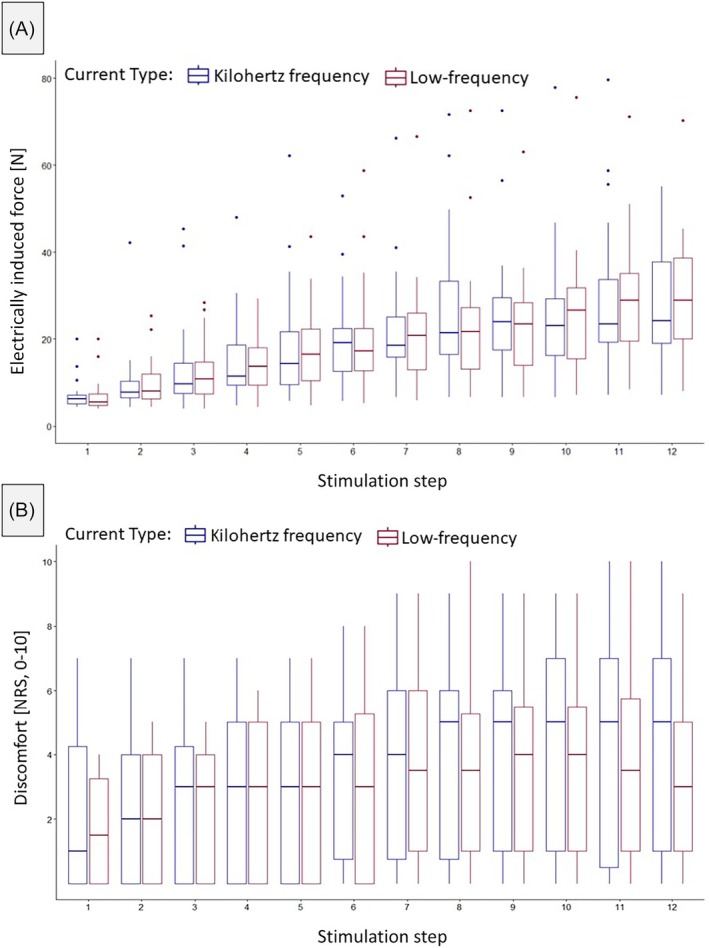
Electrically induced force [N] (A) and perceived discomfort (numerical rating scale, 0–10) (B) during the stepwise increase in stimulation intensity protocol for both current types. Data are displayed as boxplots (median, interquartile ranges, and whisker bars). NRS, numerical rating scale.

Intercept (*p* < .001) and stimulation step (*p* < .001) were significant predictors with electrically induced force increasing on average by 2.17 (1.52–2.81) [N] per step. Neither an effect for current type nor an interaction effect was found (*p* > .05). Most of the variance could be explained by the interaction between participant and current type (28.75 ± 5.36 N) followed by participant (23.28 ± 4.83 N). Full results can be found in Table 3. Descriptives are displayed in Table 2 and in Figure 5A.

MVIC and stroke type were included in the model for the covariate analysis, with MVIC showing a significant effect (*p* = .004).

### Discomfort (numerical rating scale, 0–10; secondary outcome)

Intercept (*p* < .001) and stimulation step (*p* < .001) were also significant predictors of discomfort, with an average increase in discomfort level of 0.24 (0.15–0.34) points on the numerical rating scale per stimulation step. Neither an effect of current type nor an interaction effect was found (*p* > .05). The majority of the variance was accounted for by individual participant differences (3.77 ± 1.94 points) followed by the interaction between participant and current type (1.04 ± 1.02 points). Full results can be found in Table 3. Descriptives are displayed in Table 2 and in Figure 5B.

For the covariate analysis, MVIC, stroke type, pressure pain threshold, gender, and lever arm were integrated into the model. Both pressure pain threshold (*p* = .002) and lever arm (*p* = .016) yielded significant results.

### Muscle fatigue (decrease in electrically induced force, [N]; secondary outcome)

**FIGURE 6:**
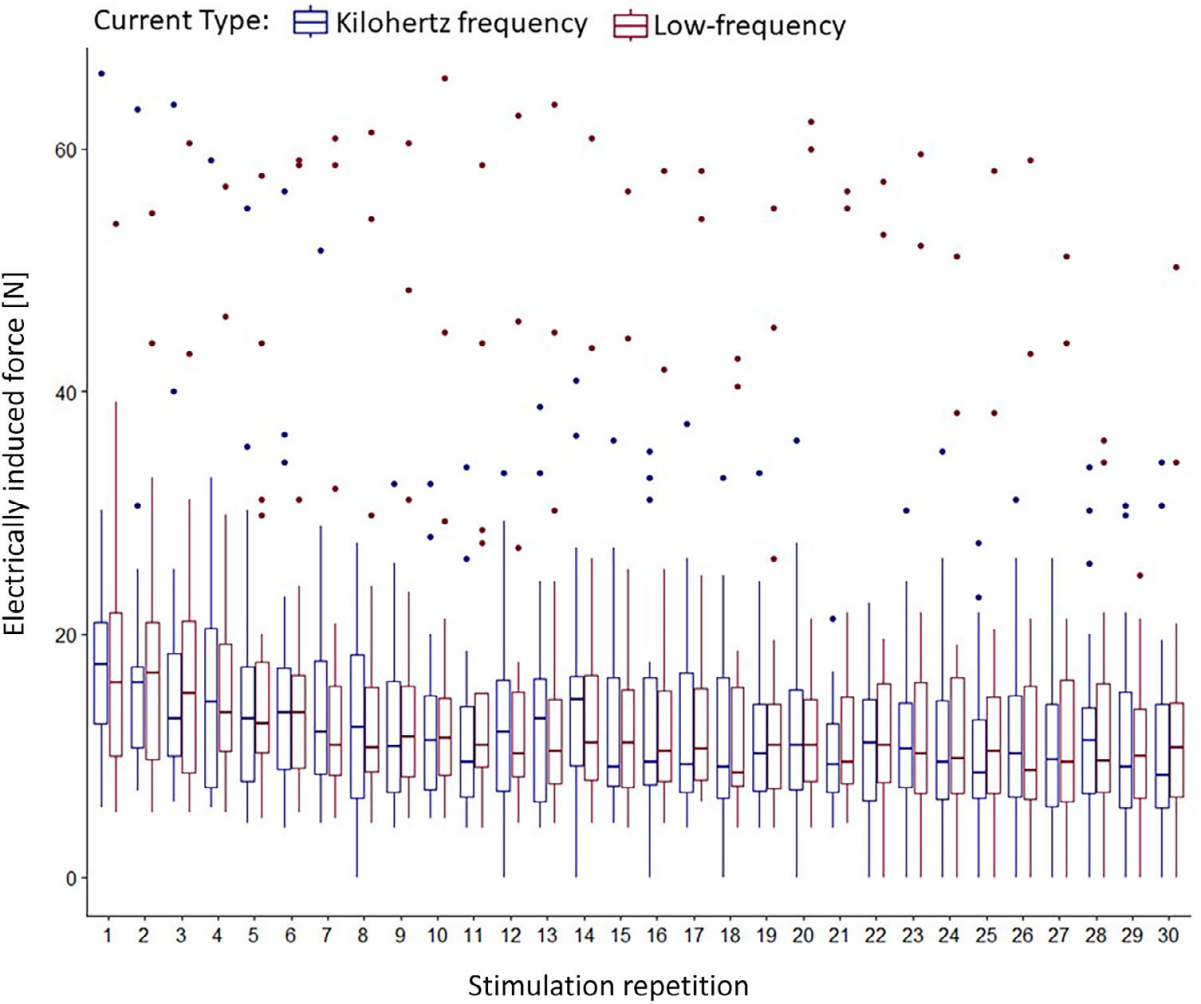
Electrically induced force [N] during the muscle fatigue protocol for both current types. Data are displayed as boxplots (median, interquartile ranges, and whisker bars).

For electrically induced force during muscle fatigue protocol, linear‐mixed‐effects model found intercept (*p* < .001) and repetition (*p* < .001) as significant predictors. Electrically induced force decreased with each repetition on average by 0.22 (−0.32 to −0.13) [N]. Neither an effect for current type nor an interaction effect was found (*p* > .05). Most variance was explained by participant (105.55 ± 10.27 N), followed by the interaction of participant and current type (80.89 ± 9 N). Full results can be found in Table 3. Descriptives are displayed in Table 2 and in Figure 6.

In the covariate analysis, the inclusion of each covariate resulted in the model failing to converge.

The complete linear‐mixed‐effects model results with covariates can be found in Table A2.

## DISCUSSION

In individuals with stroke, we observed higher stimulation efficiency with low‐frequency stimulation when compared to 1.0 kilohertz frequency stimulation of the wrist extensors. Current type had no significant effect on electrically induced force, discomfort, or muscle fatigue. Our hypotheses of comparable stimulation efficiency and less discomfort for 1.0 kilohertz stimulation are not supported for submaximal stimulation intensities. Our hypothesis that stimulation at 1.0 kilohertz frequency would result in comparable muscle fatigue and force production was confirmed. Large interindividual differences were identified in all outcomes.

### Comparison to the relevant literature: stimulation efficiency

This study provides new insights into the efficiency of wrist extensor stimulation a stroke. The efficiencies in our study are substantially lower than those found by Pinto Damo et al. in the knee extensors of healthy participants.^15^ Similar to our study, Paz et al. found higher stimulation efficiency for a 100 Hz low‐frequency stimulation of the knee extensors compared to a 2.5 kilohertz frequency stimulation modulated at 100 Hz.^26^ Although Medeiros et al. found no difference between low‐frequency and 2.5 kilohertz frequency stimulation, wider pulse duration (250 vs. 500 μs) showed greater efficiency.^16^ This was confirmed by Pinto Damo et al., even when normalized to the maximum tolerated intensity.^15^ It should be noted that the stimulation efficiency in our study is lower with kilohertz frequency stimulation, mainly due to higher stimulation intensities and not due to lower force outptut. Medeiros et al.^16^ used the maximum tolerable stimulation intensity, and although substantially higher intensities are required for kilohertz frequency stimulation, these are generally well tolerated. Consequently, the efficiency values at submaximal intensities may not hold the same significance. Furthermore, Paz et al.^26^ observed the highest efficiency with low‐frequency stimulation employing a pulse duration of 2 ms; however, they did not incorporate kilohertz frequency stimulation with a 2 ms pulse duration in their study. Similar to our findings, the study by Pinto Damo et al.^15^ showed that stimulation efficiency increased with higher stimulation intensities. In summary, it seems that efficiency increases mainly with higher stimulation intensities and longer pulse durations. However, these parameters are limited by the tolerance of the participants, as they also make the stimulation more uncomfortable.

### Torque

Laufer and Elboim,^25^ who did not include a wide pulse low‐frequency stimulation, found no difference between the current types on force production, which we can also support when compared to wide pulse low‐frequency stimulation. Ward et al.^14^ found a significantly lower force with 2.5 kilohertz frequency and short phase duration compared to 1.0 kilohertz frequency with long pulse duration and monophasic low‐frequency stimulation with long and short pulse durations. However, most of the variation and effect may be due to interindividual differences. Similarily, our study demonstrated notable interindividual variations in force output. As previously discussed, pulse duration may be crucial, as two studies^15^, ^16^ observed a significantly higher torque with a wide phase duration for the knee extensors, regardless of current type. Research is needed to assess the impact of extending the phase duration to 2 ms with 1.0 Kilohertz frequency stimulation.

### Discomfort

Our study found no effect of current type on discomfort, which is consistent with the findings of a scoping review.^13^ Only two of the included studies found higher discomfort for kilohertz frequency stimulation, and one study found higher discomfort for low‐frequency stimulation. When looking at discomfort in studies using wrist extension stimulation, one study found lower discomfort for 1.0 and 2.5 kilohertz frequency compared to monophasic low‐frequency stimulation,^14^ and one study found comparable discomfort between 2.5 kilohertz frequency and biphasic low‐frequency stimulation.^25^ Recent studies, not included in the scoping review, also report no difference between current types.^15^, ^26^, ^28^ However, Pinto Damo et al.^15^ demonstrated less discomfort with a shorter pulse duration. Given that a high level of fitness, high fat‐free mass, low systemic inflammation, and high tolerance to discomfort have been identified as strong predictors of stimulation tolerance,^29^ it is likely that the tolerance of patients with stroke could be improved by adequate stimulation familiarization. In turn, a higher tolerance of stimulation intensity will increase efficiency and force output. The successful completion of the 12 intensity steps of the step protocol by almost all patients suggests the effectiveness of our familiarization protocol.

### Muscle fatigue

In terms of muscle fatigue, two studies reported less fatigue for low‐frequency compared to kilohertz frequency stimulation, and in two other studies, fatigue was comparable between the two current types.^13^ A more recent study of the knee extensors found greater fatigue for 2.5 kilohertz frequency.^28^ Only one study examined muscle fatigue in the wrist extensors^25^ and found a significant fatiguing effect for 2.5 kilohertz frequency stimulation. The authors showed a more gradual decrease in force for the low‐frequency stimulation, whereas the kilohertz frequency stimulation showed greater fluctuations in the force profile. Similarly, in our study, force dropped to 0 during the initial 20 repetitions with 1.0 kilohertz frequency stimulation, followed by an increase in force. In contrast, with low‐frequency stimulation, force did not drop to 0 until the last third of the fatigue protocol. It is important to consider that the studies used very different fatigue protocols; 80 repetitions with the intensity inducing 20% of the MVIC^28^ and 21 repetitions with the maximum tolerable intensity.^25^ Conversely, our study used 30 repetitions at a submaximal intensity (1.25 × the first measurable force), as this protocol was deemed appropriate for the specific patient population in the pretest.

### Suggested neurophysiological mechanisms

Electrical stimulation activates the central nervous system, enhancing force generation and driving neural adaptations, while also inducing structural changes such as restoring muscle mass and improving muscle composition.^12^, ^30^, ^31^, ^32^ Notably, it significantly elevates serum levels of brain‐derived neurotrophic factor more effectively than voluntary exercise while also increasing cerebral blood flow, thereby supporting neuronal growth and brain remodeling.^33^, ^34^, ^35^

Both applied current types deliver electrical signals to motor units, facilitating muscle recruitment and cortical activation.^36^, ^37^ However, the high frequency of kilohertz frequency stimulation reduces skin resistance and can penetrate deeper tissue layers.^38^, ^39^ Its higher rate of electrical impulses, which exceeds the natural firing rate of motor neurons,^40^ may enhance motor neuron activation.^41^, ^42^ Modulating kilohertz stimulation to a physiological frequency of 50 Hz produces visible muscle contractions, likely engaging deeper muscle fibers and resulting in greater neuronal impact compared to low‐frequency stimulation. Conversely, low‐frequency stimulation may provide more targeted muscle activation, potentially offering superior strength outcomes through its specificity in muscle fiber recruitment. These distinctions underscore the unique applications of each stimulation type based on the recovery stage.

### Clinical relevance

The higher efficiency of low‐frequency stimulation at submaximal stimulation intensities and a potentially less fatiguing effect would make this current type the preferred choice for patients with stroke. However, kilohertz frequency stimulation may potentially have a higher neuronal impact. Because neuronal regulation is most active early in the recovery process following a stroke and diminishes within a short period,^43^ kilohertz stimulation may be particularly effective for early neuronal reactivation during the initial days. In contrast, low‐frequency stimulation may be better suited for promoting strength gains and driving morphological adaptations during later recovery stages (weeks to months), when tissue reorganization is more pronounced (Dobkin & Carmichael, 2016).

It is important to note that our study is a cross‐sectional investigation focusing on the acute effects of the two electrical stimulation currents, rather than the long‐term treatment effects, which remain to be investigated. These acute effects offer valuable insights for optimizing stimulation settings.^44^ This is especially relevant, as we found large inter‐individual differences highlighting the importance of individually selecting the appropriate stimulation parameters. As outlined by Maffiuletti et al., key factors influencing treatment efficacy include electrode positioning, the electrically induced force, and the perceived discomfort.^44^ Acute responses, such as force and discomfort, can be used as practical metrics to calibrate both electrode placement and stimulation parameters. Specifically, the ratio of generated force to perceived discomfort could guide the initial electrode placement, with subsequent adjustments to stimulation parameters to further enhance treatment efficacy. The stimulation parameters yielding the highest force‐to‐discomfort ratio should be used in treatment protocols, with regular assessments for monitoring and adjustments according to the patient's goals. However, this requires the use of a discomfort scale starting at 1 instead of 0. In addition, it should be noted that patients with stroke may have sensory impairments, which could affect the reliability of discomfort level reporting. Therefore, it is important to carefully monitor whether further increase in stimulation intensity leads to an increased force production without inducing co‐contractions.

Furthermore, to achieve long‐term effects, it is important to manage muscle fatigue in both types of electrical stimulation to allow for a sufficient number of repetitions, which is critical for motor relearning.^45^ This can be accomplished by introducing rest intervals. For kilohertz frequency stimulation, shorter and more frequent breaks may be required, whereas for low‐frequency stimulation, longer but less frequent breaks may be more suitable.

Although passive/cyclic stimulation was used in this study, it is essential to apply functional electrical stimulation whenever possible in in clinical practice. This involves combining electrical stimulation with functional tasks to achieve further functional improvements for patients.^5^, ^8^, ^9^, ^46^, ^47^

In the absence of voluntary movement, passive electrical stimulation can play a crucial role in developing basic motor skills, serving as a preparatory stage for more advanced motor abilities. This is particularly crucial, as the combination of severe initial impairment and limited motor improvement within the first month is a key predictor of poor long‐term motor recovery for upper limb function.^48^, ^49^, ^50^ Additionally, the basic movement of finger extension is particularly associated with better recovery of bimanual hand use.^51^ One study suggests that neuromuscular electrical stimulation may be particularly beneficial during the subacute phase for patients with severe paresis.^7^ Nevertheless, the long‐term objective, even with the use of functional electrical stimulation, should always be to enable the patient to perform movements or exercises independently.

### Limitations

Given that our study focused on individuals with stroke with an average age of 66 years, the possibility of peripheral denervation due to the stroke^52^ and age‐related degenerations^53^ cannot be dismissed. However, wrist extension was successfully induced in all participants included, suggesting intact peripheral innervation. In one participant who was excluded, peripheral deinnervation might have been present, as wrist extension could not be stimulated. Another important limitation could be the low statistical power, as only 20 instead of the originally planned 21 participants could be included in the analyses due to recruitment problems.

The small number of individuals, combined with the large number of measurements per individual, reduces the robustness of the results, limiting their generalizability. In addition, the small sample size did not allow for gender‐based analysis. For sufficient statistical power, multicenter studies should be conducted in the future. Despite instructions to refrain from upper extremity interventions on the day preceding the study and the scheduling of the second measurement session 48 hours after the first, the complexity of clinical structures made it challenging to stick to the study procedure, and this could not always be guaranteed.

Additionally, we examined only the acute effects, and a long‐term intervention study investigating the application of both current types over several weeks in relation to neuromuscular and functional recovery would be valuable.

## CONCLUSION

Low‐frequency stimulation seems to have higher stimulation efficiency but does not differ significantly from kilohertz frequency stimulation in terms of electrically induced force, discomfort, or muscle fatigue. Considering that higher intensities may be tolerated with kilohertz frequency stimulation, future studies should, from a practical point of view, include a ratio of force to discomfort as an outcome measure. Due to considerable interindividual variations and different training effects of current types, therapists should (1) consider a combination of both current types and (2) make individualized decisions regarding the choice of current. Further research is needed to explore the long‐term treatment effects of both current types.

## FUNDING INFORMATION

This research received external funding from the German Federal Ministry for Economic Affairs and Energy (Grant ZF4478606TS9).

## DISCLOSURES

The authors declare no conflict of interest.

## INSTITUTIONAL REVIEW BOARD STATEMENT

The study was conducted according to the guidelines of the Declaration of Helsinki, approved by the local Ethical Commission of the University of Applied Sciences Trier, Trier, Germany (No. 01‐2023) and registered a priori in the German Clinical Trials Register (DRKS‐ID: DRKS00030666).

## INFORMED CONSENT STATEMENT

Informed consent was obtained from all participants involved in the study.

## Supporting information


**Supporting Information.** Appendix.

## Data Availability

Data are available upon reasonable request from the corresponding author.
